# Evaluation of axillary lymph nodes in breast cancer patients with clinically negative axilla using contrast enhanced ultrasonography

**DOI:** 10.1186/s12957-024-03419-0

**Published:** 2024-08-07

**Authors:** Roshit Jain, Rahul Khanna, Ashish Verma, Shashi Prakash Mishra, Ram Niwas Meena, Seema Khanna, Siddharth Khanna

**Affiliations:** 1 Department of General Surgery, Institute of Medical Sciences, Banaras Hindu University, Varanasi, 221005 India; 2 Department of Radiodiagnosis, Institute of Medical Sciences, Banaras Hindu University, Varanasi, 221005 India; 3 Dept of Ophthalmology, Aligarh Muslim University, Aligarh, 202002 India

**Keywords:** Breast cancer, Contrast enhanced ultasonogram

## Abstract

Contrast enhanced ultrasonography enables dynamic evaluation of the microvasculature down to the capillaries when using high resolution ultrasound probes. It’s application in the evaluation of axillary lymph nodes in breast cancer patients with clinically negative axilla has been studied in 42 patients. The results of pre operative CEUS evaluation was correlated with histopathology status of axillary nodes after the harvesting of nodes during modified radical mastectomy or sentinel node biopsy. Heterogeneous enhancement with micro bubbles of the axillary nodes was found to be the most distinguishing criteria for malignant nodes.

## Background

The status of draining lymph nodes, specially the axillary lymph nodes is an important prognostic factor with reference to recurrence and survival in breast cancer. It is also an important predictive factor for determining the need and type of adjuvant therapy.

Over the last 30 years, assessment of axillary nodes has undergone significant changes. Clinical examination of the axilla has been found to be unreliable with under estimation rates of 30% for malignant nodes, especially in obese patients [1]. Sentinel lymph node biopsy was introduced in 1990s and became the standard surgical method of staging the axilla in clinically node negative women [2]. However, the results of sentinel node biopsy status are obtained intra operatively and not available before the operation for treatment planning and patient counseling.

Routine pre-operative ultrasound evaluation of the axilla is now recommended guideline in breast cancer patients. The ultrasound characteristics of a metastatic lymph notes are fairly well defined. They include parameters such as long to short axis ratio, irregular margins, hypoechoic centers, peripheral halos and absent hilum [3].

It has been suggested that effectiveness of conventional ultrasound can be improved by contrast enhancement. The rational being that peritumoural injection of microbubble contrast would be better able to identify the sentinel axillary node for assessment and perhaps a guided FNAC or core needle biopsy [4].

Therefore, the present study was undertaken with the aim of determining parameters which can differentiate metastatic from normal lymph nodes in breast cancer patients using contrast enhanced ultrasound (CEUS) evaluation of the axilla.

## Materials and methods

Over a 4 years period from February 2019 to January 2023, 42 patients of cytologically or histologically proven breast cancer were included in the study. Only patients in whom there was no palpable lymph node in the axilla were included. The exclusion criteria for patient recruitment were:-.

1. Palpable axillary lymph nodes.
2. Pregnancy.
3. Previous treatment (chemotherapy or radiotherapy or surgery).
4. Patients with right to left cardiac shunts, pulmonary hypertension, uncontrolled hypertension or adult respiratory distress syndrome or hypersensitivity to the drug.

All breast cancer patients underwent a detailed history and clinical examination which were recorded in their case sheets. Tissue diagnosis was obtained by FNAC or core needle biopsy. A total of 118 breast cancer patients were seen in the study period of which 76 were excluded on the basis of exclusion criteria described above. Thus 42 patients were available for the purpose of the study.

The patients underwent a conventional ultrasound of both breast and axilla, followed by a contrast enhanced ultrasound (CEUS) of the diseased breast only. For the purpose of CEUS, 0.2 to 0.4 ML of contrast agent (Perflutren lipid microsphere) was injected intradermally in the upper outer quadrant at the areolar edge of the involved breast. The standard dose of ultrasonography contrast media is 10 µL/Kg, accordingly the dose titrates to about 0.5 to 0.7 mL for most of our patients. However in our experience for small parts CEUS using high frequency transducer this dose leads to oversaturation of images. A dose of 0.2 to 0.4 mL was found optimum and therefore chosen after performing a few pilot cases. Gentle massage of the area was done to stimulate lymph flow towards the axilla. Contrast pulse sequence ultrasound was used to visualize the lymph channels which drained into the axilla, where the contrast medium accumulates in the first draining lymph-node. The CUES assessment of the lymph node was done at 2, 4 and 6 min after injection of the contrast.

The patient was then taken up for upfront surgery which was either (a) Modified radical mastectomy: 24 patients, (b) Breast conservation surgery with axillary clearance (for patients unwilling for post operative radiotherapy treatment): 11 patients, (c) Breast conservation surgery with sentinel lymph node biopsy: 7 patients. The histopathological report of the axillary lymph nodes harvested during surgery was subsequently correlated with the findings of CEUS of the axilla.

## Results

A total of 118 breast cancer patients were evaluated in the 4 years study period of which 76 did not fulfill requirements of the study as per the exclusion criteria. Thus there were 42 patients recruited for the purpose of the study. The mean age of the recruited patients was 53 years (range 39 to 72 years.) The TNM staging is shown in the Table 1. It may be noted that the stage grouping is that of only patients recruited for the study who had to be cN0 to fulfill the inclusion criteria. It does not reflect the patient profile of breast cancer patients visiting our department. As part of our metastatic work-up protocol all patients underwent X ray of chest and ultrasonogram of the abdomen. No evidence of metastasis was found on clinical evaluation or imaging studies among the 42 patients recruited for the study.

**Table 1:** Stage grouping of breast cancer patients recruited for assessment by CEUS

Stage grouping as per TNM system	No. of patients
T1N0	23 (55%)
T2N0	14 (33%)
T3N0	5 (12%)
Total	42

On CEUS, the parameters evaluated in the axillary nodes were -.

1. Enhancement pattern (homogenous/ heterogenous/ no uptake).
2. Enhancement intensity (no uptake/ Isoenhancement/ hypodense enhancement/ hyperdense enhancement)(The intensity of enhancement was defined according to visual perception of contrast uptake in the lymph node in comparison to adjacent tissue).
3. Time taken to achieve Peak enhancement (No Uptake/ early/ synchronous/ delayed)(The ultrasound machine shows the time in milliseconds after initiating the cine-loop. Early, synchronous/delayed enhancement was in comparison to adjacent tissue).
4. Direction of contrast uptake (centripetal/ centrifugal) (Baseline sonography helped us label the lymph node to be investigated using CEUS. Following this the uptake of contrast microbubbles within specific node as centripetal(from out to in) or centrifugal (from in to out) in direction).
5. Presence/ absence of radial feeding vessel (We did not try to separate blood vessels from lymphatic vessels as for the purpose of CEUS both play a role, hence visualization of both remain equally important).

**Table 2:** Enhancement characteristics of axillary lymph nodes on CEUS in breast cancer patients with clinically N0 status (*n* = 42) correlated with the histological reports of axillary nodes after surgery

CEUS Parameters	Benign (*n* = 27)	Malignant (*n* = 15)	*p*-Value		
**1)Enhancement Pattern**					
Homogenous	21(77%)	02(14%)	**0.000278**		
Heterogeneous	4(15%)	10(67%)			
No Uptake	2(8%)	3(20%)			
**2)Enhancement Intensity**					
No Uptake	2(8%)	3(20%)	**0.026552**		
Isoenhancement	14(52%)	2(14%)			
Hypodense enhancement	6(22%)	2(14%)			
Hyperdense enhancement	5(18%)	8(54%)			
**3) Enhancement time taken**					
No Uptake	2(8%)	3(20%)	**0.113683**		
Early	3(11%)	5(33%)			
Synchronous	16(59%)	4(26%)			
Delayed	6(22%)	3(20%)			
**4)Enhancement Direction**					
Centripetal	21(77%)	11(73%)	**0.523103**		
Centrifugal	4(15%)	1(7%)			
**5)Radial or feeding vessel**					
Present	2(8%)	3(20%)	**0.156788**		
Absent	23(85%)	9(60%)			

*Please note that in parameter number 4 (enhancement direction) and 5 (radial feeding vessel), the numbers do not add up to 100% because patients with no contrast uptake were not included in these two groups

**Fig. 1:**
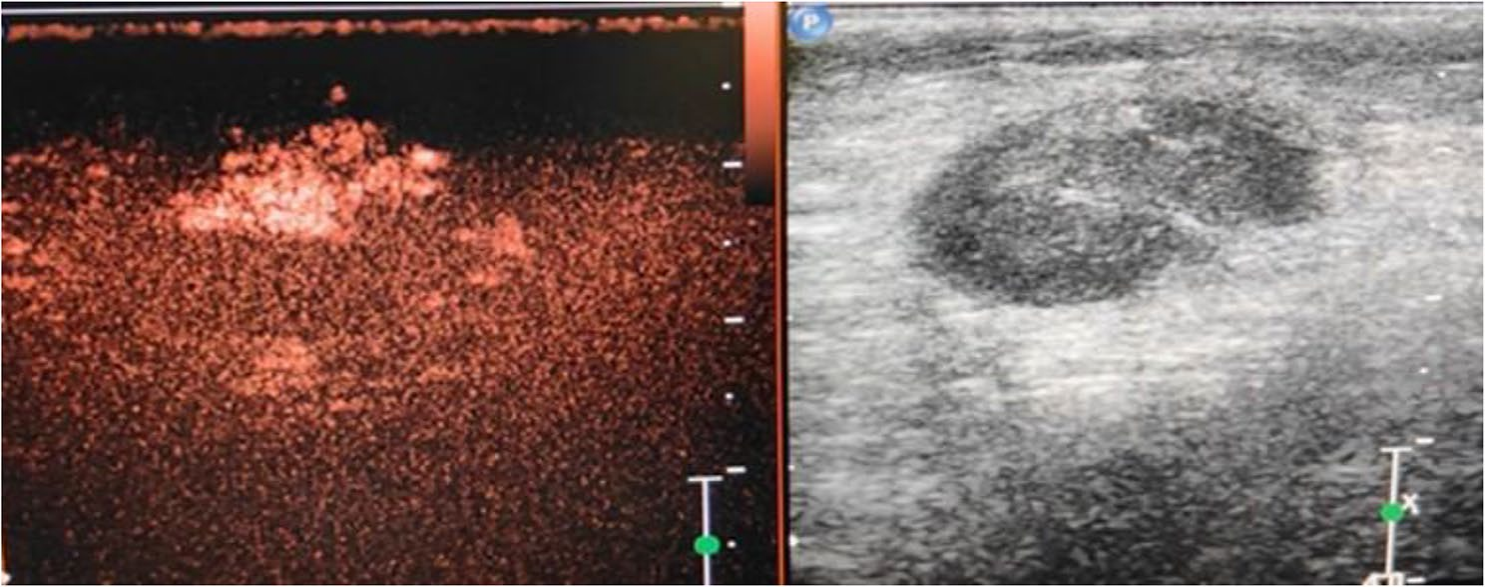
Heterogenous enhancement of axillary lymph node on CEUS in a patient with a Breast Carcinoma:

**Fig. 2:**
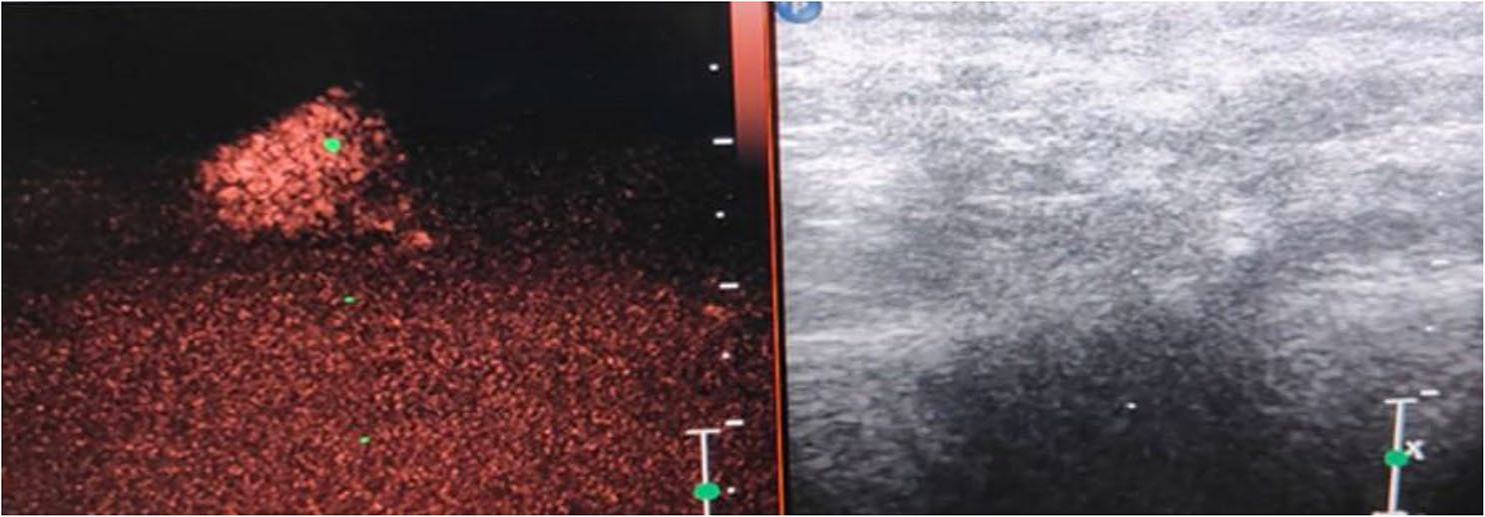
Homogenous enhancement of axillary lymph node on CEUS in a patient with Fibroadenoma:

On comparison of pre-operative CEUS findings of the axillary lymph nodes with the final histopathology of the nodes obtained at axillary clearance or sentinel lymph node biopsy it was noticed that the only parameter to achieve a significant difference (*p* < 0.05) between benign and malignant nodes was the contrast enhancement pattern. Malignant nodes had a greater tendency to demonstrate heterogeneous enhancement compared to benign nodes (67% vs. 15%) (Fig. 1). Benign lymph nodes had a greater propensity for homogenous contrast enhancement (77% vs. 14%) (Fig. 2). Absent update of contrast the was more common in malignant nodes (20% vs. 8%) (Table 2).

With regards to intensity of contrast enhancement, isoenhancement was more often found in benign as compared to malignant nodes (52% vs. 14%). Hyperdense enhancement was more common with malignant nodes (54% vs. 18%).

Significant difference was not observed with reference to time taken to achieve peak enhancement. Peak enhancement was measured as the screen pixel value in the phase with maximum visually perceptible enhancement after injection of the contrast agent. Generally it was found that early enhancement was more often seen in malignant nodes (33% vs. 11%) while synchronous contrast enhancement of nodes as compared to surrounding tissue was a predominant feature of benign lymph nodes (59% vs. 26%). Synchronicity was defined in comparative terms between target nodes and not between nodes and surrounding structures. This gave a perception of earlier contrast uptake in certain nodes while delayed in others.

Significant differences in contrast enhancement pattern between benign and malignant nodes was not observed with reference to enhancement direction (centripetal/centrifugal) or the presence or absence of radial or feeding vessel.

## Discussion

CEUS utilizes the injection of a contrast agent (Perflutren liquid microsphere in our study) in the involved breast. The agent is carried by the lymphatic channels to the first draining lymph-node in the axilla. Thus CEUS works on the same principles as used for intra-operative identification of sentinel lymph nodes using vital blue dye or a radio-isotope. Therefore the lymph node identified in the axilla on CEUS should be considered as the sentinel node for all practical purposes.

Perflutren comprises lipid coated microspheres filled with octafluoropropane gas. When exposed to ultrasound waves, the microspheres resonate and ‘echo’ strong signals back to the ultrasound machine. the other agent that has been used for purpose of CEUS is sulphur hexafluoride gas suspended as microbubbles in a liquid. The gas trapped in microbubbles is not soluble in body fluids or water. It is removed naturally from the blood through respiration.

The pattern of contrast enhancement, intensity, speed of enhancement and also washout are well defined parameters used for characterization of liver tumors and other solid organ tumors. Generally malignant tumors and metastasis in liver exhibit the following characteristics. Hypervascular metastasis and hepatocellular carcinoma exhibit hyperenhancement during the arterial phase while some metastasis appears as hypoenhanced or demonstrate a rim like enhancement in the arterial phase. Larger malignant lesions may show a disorganized internal vasculature which becomes more visible on CEUS studies than it does on non contrast enhanced CT or MRI [5].

Although there is a significant amount of overlap in enhancing characteristics of benign and malignant lesions, it is generally found that malignant lesions demonstrate heterogeneous or peripheral hyper enhancement with centripetal filling. Further as a consequence of neo angiogenesis and intra-tumoral arterio-venous shunting malignant lesions have a rapid wash in and wash outs [6]. A homogeneous enhancement is a reliable indicator of a benign breast lump.

In our study on the utility of CEUS for assessment of nodes in clinically negative axilla of breast cancer patients, we have attempted to extrapolate the previously described contrast enhancement patterns seen in liver tumors. Our study has shown that malignant lymph nodes had a greater propensity for heterogenous enhancement compared to benign nodes (67% vs. 15%). Three out of fifteen malignant lymph nodes (20%) did not exhibit contrast uptake probably due to necrosis in the node. Benign lymph nodes had more chances of being isoenhancing with comparison to surrounding breast parenchyma compared to malignant nodes (52% vs. 14%). As far as time taken to enhance, enhancement direction (centripetal or centrifugal) and feeding vessel we could find no difference between benign and malignant lymph nodes.

CEUS with conventional ultrasonogram has better diagnostic performance than conventional ultrasonogram alone. Both quantitative and qualitative CEUS parameters have been used to distinguish benign from malignant breast masses. Luo et al. (2016) described 10 different CEUS parameters and reported 3 different malignant predictive models and 3 benign models [7]. The parameters studied were contrast enhancement, direction of contrast uptake and speed of contrast uptake [8]. 

Quantitative parameters in CEUS which favor a malignant diagnosis are shorter time taken by micro-bubbles to reach peak enhancement and a higher peak intensity [9]. These findings are a consequence of neo angiogenesis, arterio-venous connections and higher micro vessel density seen in malignant lesions. Although the difference is statistically significant, there is a great deal of overlap and a clear cut-off value cannot be assigned for clinical practice.

Just like most of ultrasound reporting, interpretation of CEUS images is operator dependent. In hands of experienced sonologists CEUS has potential just like MRI to provide quantitative parameters as well as functional assessment through post processing including a time intensity curve [10]. CEUS has the potential to downgrade BIRADS 4 lesions using parameters such as presence or absence of enhancement, pattern of enhancement, mass margins and shape [11]. Benign lesions are more likely to be isoenhancing and have circumscribed margins while malignant lesions will demonstrate hyper enhancement (or no enhancement in case of necrosis) and have indistinct or reticulated margins. It has been proposed that oval masses with homogeneous enhancing pattern and circumscribed margins may be spared a biopsy procedure.

As mentioned above we believe that the axillary lymph node which is the first to take up micro bubbles on CEUS should be considered as the sentinel lymph node. If the ultrasound features are suspicious a guided FNAC can be obtained from this node. This will be following the concepts and principles of intra-operative harvesting and evaluation of the sentinel node. The added advantage of our proposed technique is that a sentinel node tissue diagnosis would be available before the surgery. This will greatly aid in treatment planning as well as patient counseling. The caveat to our proposal is that in patients who have a positive for malignancy report in pre-operative CEUS guided FNAC should be treated as such. However those who have a negative for malignancy report should be treated as an equivocal report and should be managed accordingly with a conventional intra-operative sentinel node biopsy if indicated.

Sever AR et al. used a similar technique of peri-areolar intradermal injection of micro-bubble contrast agent. Breast lymphatics were visualized by sonography and followed to the axilla to identify sentinel lymph node. A guidewire was deployed at the same time under ultrasound guidance to localize the sentinel node. The next day the patient underwent standard tumor excision and sentinel node biopsy using conventional radioisotope and blue dye technique. Among the fourteen patients found to have metastasis in sentinel lymph node it was found that the sentinel node was correctly identified by the CEUS and localized with guidewire before surgery [12]. 

Contrast Enhanced Ultrasound evaluation can be performed within 90 s. The microbubble contrast is well tolerated with few side effects. It can be performed in patients with renal failure who cannot undergo contrast CT scan. Also it can easily be done in those with metallic implants or pacemakers when MRI is contraindicated or those who suffer from claustrophobia which can occur in CT scan or MRI machines. Thus our results show that CEUS can significantly improve the diagnostic value of ultrasound evaluation of the axilla in breast cancer patients.

## Data Availability

All data regarding the study can be contacted by Dr Roshit Jain, Department of General Surgery, Institute of Medical Sciences, Banaras Hindu University, Varanasi, 221005, India. (Email –roshitjain478@gmail.com)
